# Analysis of Genotype 1b Hepatitis C Virus IRES in Serum and Peripheral Blood Mononuclear Cells in Patients Treated with Interferon and Ribavirin

**DOI:** 10.1155/2014/175405

**Published:** 2014-07-03

**Authors:** Iwona Bukowska-Ośko, Kamila Caraballo Cortés, Agnieszka Pawełczyk, Rafał Płoski, Maria Fic, Karol Perlejewski, Urszula Demkow, Hanna Berak, Andrzej Horban, Tomasz Laskus, Marek Radkowski

**Affiliations:** ^1^Department of Immunopathology of Infectious and Parasitic Diseases, Medical University of Warsaw, 3c Pawińskiego Street, 02-106 Warsaw, Poland; ^2^Department of Medical Genetics, Medical University of Warsaw, 3c Pawińskiego Street, 02-106 Warsaw, Poland; ^3^Department of Laboratory Medicine and Clinical Immunology of Developmental Age, Medical University of Warsaw, 24 Marszałkowska Street, 00-576 Warsaw, Poland; ^4^Hospital for Infectious Diseases, 37 Wolska Street, 01-201 Warsaw, Poland

## Abstract

Hepatitis C virus (HCV) highly conserved IRES (internal ribosome entry site) sequence, localized within the 5^′^-untranslated region (5^′^UTR), may determine viral properties like replication efficiency and cell tropism. The aim of the present study was to characterize newly emerging 5^′^UTR variants in serum and peripheral blood mononuclear cells (PBMC) in chronic hepatitis C patients treated with interferon (IFN) and ribavirin and to identify their effect on IRES secondary structures. The study group consisted of 87 patients infected with genotype 1b from whom serum and PBMC samples were collected at 9 time points (before, during, and after treatment). New 5^′^UTR variants developed in 9 patients. Out of the overall 14 new variants, 9 (64%) were found in PBMC. HCV variants with decreased thermodynamic stability were identified only in PBMC and C183U mutation was the most common one in this compartment. In conclusion, antiviral treatment may favor emergence of new 5^′^UTR variants both in blood and in PBMC compartments. However, variants developing in the latter compartment were predicted to have lower thermodynamic stability of the IRES secondary structures compared to serum strains. C-U change in position 183, which has not been described previously, might indicate viral adaptation to lymphoid cells.

## 1. Introduction

Hepatitis C virus (HCV) displays significant molecular variability and circulates in the infected host as a heterogenous population referred to as* quasispecies *[1, 2]. This dynamic population of closely related but not identical variants could play a significant role in immune evasion, resistance to antiviral therapy, and adaptation to the cells of the immune system [1, 3, 4].

IRES (internal ribosome entry site) sequence is localized between nucleotide positions 40 and 372 and partly overlaps with both the 5′untraslated region (5′UTR) and the open reading frame [5]. It forms a secondary structure of high stability containing four domains [2, 5–7].

Domain II is crucial for RNA replication and translation [5]. Mutations within this region may decrease translation efficiency [5]. The most complex part of IRES is domain III that is composed of branched structures (hair pins) IIIabcde with inner loops inside some of them; however, it represents the most stable fragment of HCV genome with regard to nucleotide sequence and secondary structure. Domain III participates in the maintenance of the whole IRES secondary structure stability [5]. It binds the 40S subunit and interacts with the eukaryotic initiation factor eIF3 and ribosomal proteins thus playing a critical role in translation [8].

The AUG start codon at position 342 and the first 11 nucleotides of the open reading frame (ORF) are localized in the last IRES domain IV [9, 10].

In addition to its role in the translation initiation, 5′UTR is likely to confer cellular tropism, as specific mutations are often identified in variant isolated from extrahepatic compartments such as PBMC, lymphoid system, brain, and bone marrow [1, 11–14].

The aim of the present study was to characterize polymorphism of IRES domains II and III in serum and peripheral blood mononuclear cells (PBMC) in chronic hepatitis C patients treated with interferon (IFN) and ribavirin and to identify their effect on IRES secondary structures.

## 2. Materials and Methods

The study group consisted of 87 patients monoinfected with HCV genotype 1b who were treated with PEG-IFN*α* (PEGASYS ROCHE or PEGINTRON SCHERING) and ribavirin (COPEGUS ROCHE or REBETOL SCHERING) for 48 weeks. The study was approved by the Internal Review Board at the Warsaw Medical University (reference number KBO/23/09), and each patient signed an informed consent form. There were 44 women and 43 men; their mean age was 44 years (range from 19 to 69). None of the patients has been previously treated for hepatitis C and none has had history of decompensated liver disease. Furthermore, all patients were negative for anti-HIV. The sustained virological response (SVR) rate among our patients was 67%.

Serum and PBMC samples were collected: before treatment (baseline), during treatment (weeks 4, 6, 8, 16, 24, and 48), and after treatment (weeks 60 and 72).

Sera were isolated 2 hours after blood drawing and PBMC were isolated by density gradient centrifugation [15]. Both sera and PBMC samples were immediately frozen and kept at −80°C until analysis. RNA was extracted from 3 × 10^6^ to 1 × 10^7^ cells and from 250 *μ*L of serum by Chomczynski method. One-quarter (1/4) of this RNA solution was used for each RT-PCR reaction [16].

5′UTR HCV RNA was amplified as described elsewhere [12]. Amplified RT-PCR products were first screened by SSCP (single-strand conformation polymorphism) [17], and whenever band pattern indicated sequence change, the samples involved were sequenced after initial cloning.

PCR products were cloned using TA Cloning Kit (Invitrogen). Plasmids were purified with Quick Plasmid Miniprep Kit (Invitrogen) and sequenced using Applied Biosystems 3130 Genetic Analyzer. Sequences were analyzed using the MEGA 5.0 program [18].

Prediction of IRES domains II and III secondary structures and thermodynamic stability was performed separately for each domain using MFOLD 3.2 program http://mfold.rna.albany.edu [19].

## 3. Results

5′UTR viral sequences were amplified from sera and PBMC from all 87 patients and first analyzed by SSCP. In 9 patients, the SSCP band pattern changes during treatment, and the exact nature of these changes was further analyzed by cloning and sequencing.

The characteristics of patients with and without 5′UTR changes are summarized in Table 1.

Altogether, 14 newly emerging HCV variants were identified: 10 developed during treatment and four appeared only after the end of therapy. Seven of these emerged in PBMC (50%), five (36%) emerged in serum, and two (14%) emerged in both serum and PBMC (Table 2).

**Figure 1:**
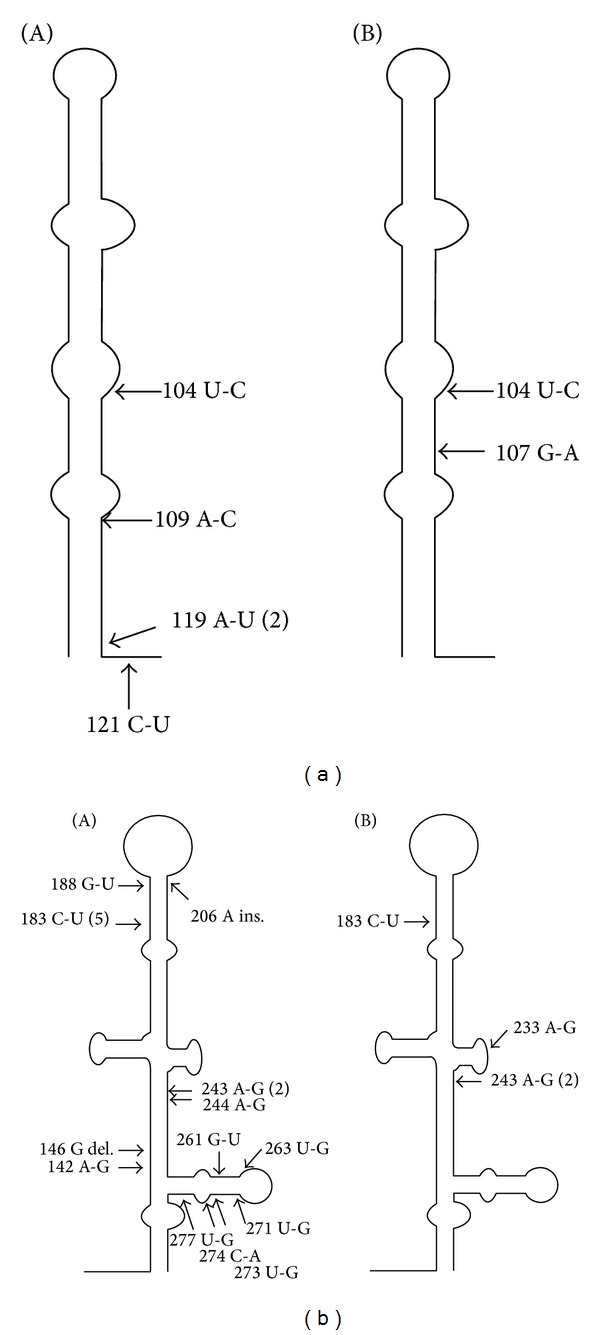
Distribution of detected mutations in IRES domains II (I) and III (II) secondary RNA structure in newly emerging variants appearing during treatment (a) and after completing therapy (b). Arrows indicate localization of nucleotide changes; number of variants is provided in brackets; del., deletion; ins., insertion.

Within the 14 new variants there were 22 point mutations distributed in domains II and III. Most of the observed changes were substitutions 20/22 (91%); in the remaining two, there was one deletion and one insertion (Table 3, Figure 1).

Sixteen (73%) of the 22 mutations were localized in domain III including seven in domain IIId (G261U, U263G, U273G, C274A, U277G, U271G, and G271U), two within domain IIIa (one substitution A142G and one G deletion at position 146), four in domain IIIb (C183U, G188U, A233G, and one insertion 206A), and three in domain IIIc (A243G, G243A, and A244G). The remaining six mutations emerged in domain IIb (U104C, C104U, G107A, and A109C) and in ssRNA junction between domains II and III (A119U and C121U) (Table 3).

Analysis of the localization of these mutations in the predicted IRES secondary structure showed that 18/22 (82%) occurred in the paired regions of both analyzed domains, whereas two were observed in domains II and III loops at positions 109 and 183, respectively, and two were observed in ssRNA regions (Figure 1).

### 3.1. Compartmentalization of New Variants

Eleven IRES mutations were detected exclusively in variants amplified from PBMC (U104C, A109C, A142G, 146Gdel, G188U, 206Ains, A233G, A243G, A244G, G261U, and G271U), whereas three were identified only in serum (C104U, G107A, and C121U). Eight substitutions were found in both serum and PBMC (A119U, C183U, G243A, U263G, U271G, U273G, C274A, and U277G) (Table 3).

Viral variants appearing in PBMC have had more changes than those appearing in serum. Thus, 5 serum-derived variants (Pt. 1, 2, 7, and 9) contained only a single mutation when compared to the strains in baseline population. In contrast, 6 out of 7 newly emerging variants in PBMC (Pt. 3 and 6–9) contained 2 to 5 mutations per variant (Table 2). One quasispecies variant emerging in both compartments (Pt. 5) contained 5 changes with respect to the initial sequence.

The C183U substitution was the most common mutation as it was found in 6 variants present in four patients (Pt. 3 and 6–8) (Tables 2 and 3). The C183U substitution was identified in five PBMC-derived variants and in one sequence present in serum. Another change (A119U) was detected in two different variants (Table 3).

All new variants isolated from PBMC have had changes at positions 204 and 243: C204 and A243 were found in two variants, C204 and G243 were found in five variants, U204 was found in one variant, and G243 was found also in one variant (Table 3). U104C and A243G variants appeared during therapy, but after discontinuation of treatment the sequence reverted back to the original (C104U and G243A).

### 3.2. Thermodynamic Stability of the RNA Secondary Structure

The Gibbs minimum free energy values (Δ*G*), which characterize the stability of RNA secondary structures, were compared between newly appearing HCV variants and the baseline viral strains (Table 3). Changes in Δ*G* were observed in eight patients altogether. Variants with decreased stability of the II and/or III domains developed in three patients, increased stability variants appeared in another three, and in one patient variants with decreased and increased stability developed simultaneously. Furthermore, in one patient, opposite changes in domains II and III stability were present within the same viral variant.

New HCV variants manifesting increase in predicted IRES structure stability appeared in both serum (three variants) and PBMC (four variants). However, variants with decreased thermodynamic stability were identified exclusively in PBMC (three variants).

Mutations affecting IRES secondary structure stability were localized both in paired RNA regions and in loops (Figure 1). Changes within ssRNA segments (nt 119 and nt 121) did not affect Δ*G* value. The most significant impact on IRES secondary structure showed C-U substitution at nucleotide 104 and G deletion at nucleotide 146, which destabilized paired region of domains IIb and IIIa, respectively. Mutations G-U at position 188, 261, or 271 disrupted RNA pairing and induced additional loop formation. Additional connection in loop (nt 183) and substitutions U-G at positions 263, 271, 273-4, and 277 increased IRES stability.

## 4. Discussion

While HCV is mainly hepatotropic, cells of the lymphoid system constitute a secondary site of replication and lymphotropic variants often differ from those circulating in blood [7, 12, 13, 20, 21]. Several studies have shown that sequence changes within the 5′UTR affect the stability of secondary RNA structures and that they affect viral translation and replication efficiency; in addition, they are likely to determine viral tropism to particular cell compartments [2, 3, 5–7, 14, 22].

The aim of the present study was to identify and characterize newly emerging 5′UTR variants in PBMC and blood of patients undergoing antiviral treatment with pegylated interferon and ribavirin. In our study, the majority of the newly emerging variants (64%) were localized in PBMC, confirming this compartment as an independent site of replication and suggesting that it is under immune pressure related to treatment.

Interestingly, all newly emerging variants with decreased IRES stability were localized in PBMC. It was previously reported that “lymphotropic” HCV variants may demonstrate impaired translation efficiency, which could be an unintended consequence of viral adaptation mutations to different cells [14]. However, lower translation efficiency could confer its own benefits, particularly in the setting of treatment-related immune pressure, as it would lower the expression of viral proteins on infected cells and thus facilitate viral survival and latency. Whether the mutations developed* de novo* or were already present and simply became dominant once the major variants were suppressed by treatment is unclear.

The decrease in viral load during treatment could impede the detection of minor variant. However, we did not see any differences in viral loads between patients with and without changes in viral sequence.

Previous studies demonstrated that the apical part of domain III is essential for effective HCV translation. Due to binding of the eukaryotic translation factor 3 (eIF3) and 40S ribosomal subunit, it positions viral RNA and enables 80S complex formation on the IRES [23, 24]. A recent study found that the competition between HCV domain III and eIF3 for binding with 40S subunit may result in the reduction of 43S complex formation and may thus favor translation of HCV mRNAs [25]. We did not find any variants with changes within the eIF3 binding sites in domain III. However, the relevance of identified mutations, especially at position 183, for eIF3 interaction and translation cannot be excluded.

## 5. Conclusion

In conclusion, HCV 5′UTR variants emerging in PBMC compartment during antiviral treatment are characterized by higher number of nucleotide changes and lower thermodynamic stability compared to serum strains. Nucleotide changes in position C183U might indicate viral lymphoid tropism.

## Figures and Tables

**Table 1:** Some characteristics of the patients with and without 5′UTR changes during treatment.

	Patients with 5′UTR changes (*n* = 9)	Patients without 5′UTR changes (*n* = 78)
Number of SVR patients	2 (22%)	56 (72%)
HCV viral load at baseline IU/mL (×10^4^)∗	181,6 ± 151,9 (range 36,6–281,0)	121,6 ± 125,4 (range 1,7–409,0)
HCV viral load at 12 weeks IU/mL (×10^2^)∗	223,8 ± 669,9 (range 0–2010,0) (*n* = 3)	7,6 ± 28,7 (range 0–209,0) (*n* = 15)
Number of patients with HCV RNA in PBMC at week 72	7 (78%)	21 (30%)
ALT (IU/L)∗	96,9 ± 63,4	107,5 ± 53,3

SVR, sustained virological response; PBMC, peripheral blood mononuclear cell; ALT, alanine transaminase; *n*, number of patients.

∗Mean ± SD.

**Table 2:** Distribution of mutations developing in the 5′untranslated region of HCV in serum and PBMC from patients treated with pegylated interferon and ribavirin.

Position	Mutation	Domain	Number of patients		Number of variants	
			SVR+	SVR−	PBMC	Serum
104	U-C	IIb		1	1	
104∗	C-U	IIb	1			1
107∗	G-A	IIb		1		1
109	A-C	IIb		1	1	
119	A-U			2	1	1
121	C-U			1		1
142	A-G	IIIa		1	1	
146	G del	IIIa		1	1	
183	C-U	IIIb	1	3	4	1
183∗	C-U	IIIb	1		1	
188	G-U	IIIb		1	1	
206	A ins	IIIb		1	1	
233∗	A-G	IIIb	1		1	
243	A-G	IIIc		1	1	
243∗	G-A	IIIc		1	1	1
244	A-G	IIIc		1	1	
261	G-U	IIId		1	1	
263	U-G	IIId		1	1	1
271	G-U U-G	IIId		2	2	1
273	U-G	IIId		1	1	1
274	C-A	IIId		1	1	1
277	U-G	IIId		1	1	1

SVR+, sustained virological response; SVR−, no sustained virological response; PBMC, peripheral blood mononuclear cell.

∗Mutations were detected after the end of therapy (weeks 60 and 72).

**Table 3:** Analysis of HCV 5′UTR mutations developing during treatment with pegylated interferon and ribavirin.

Patient	Time point of new variant appearance (weeks)	Mutation	Position	Region	SVR	Localization		Effect on Δ*G* (kcal/mol)∗∗
						PBMC	Serum	
1∗	60	C-U	104	IIb	+		1	Increased

2	8	A-U	119	ssRNA	−		1	No effect
	24	C-U	121	ssRNA	−		1	No effect

3	24	A-U	119	ssRNA	−	1		Increased
		C-U	183	IIIb				
		A-G	244	IIIc				
		G-U	261	IIId				
		G-U	271					

4	4	A-G	142	IIIa	−	1		Decreased

5	8	U-G	263	IIId	−	1	1	Increased
		U-G	271					
		U-G	273					
		C-A	274					
		U-G	277					

6∗	60	C-U	183	IIIb	+	1		Decreased
		A-G	233	IIIc				

7	8	C-U	183	IIIb	−		1	Decreased
	8	C-U	183	IIIb		1		Increased
		G del	146	IIIa				

8	16	C-U	183	IIIb	−	1		Decreased
		A-G	243	IIIc				
	24	A-C	109	IIb		1		No effect
		C-U	183	IIIb				Decreased
		A-G	243	IIIc				
		U-C	104	IIb				

9∗	16	G-U	188	IIIb	−	1		Decreased
		A ins.	206	IIIb				Increased
				IIIc				
		A-G	233	IIIb				
		G-A	107	IIIc				
				IIb				
		G-A	243	IIIc				
	60						1	No effect
	72					1	1	No effect

SVR, sustained virological response; PBMC, peripheral blood mononuclear cell.

∗Novel variants emerging after treatment (weeks 60 and 72).

∗∗Increased Δ*G* implies lower stability, whereas decreased Δ*G* entails higher stability.

## References

[1] Farci P (2011). New insights into the HCV quasispecies and compartmentalization. *Seminars in Liver Disease*.

[2] Friebe P, Lohmann V, Krieger N, Bartenschlager R (2001). Sequences in the 5′ nontranslated region of hepatitis C virus required for RNA replication. *Journal of Virology*.

[3] Soler M, Pellerin M, Malnou CE, Dhumeaux D, Kean KM, Pawlotsky J (2002). Quasispecies heterogeneity and constraints on the evolution of the 5' noncoding region of hepatitis C virus (HCV): relationship with HCV resistance to interferon-*α* therapy. *Virology*.

[4] Bukowska-Osko I, Radkowski M, Pawełczyk A (2013). Hepatitis C virus 5′ untranslated region variability correlates with treatment outcome. *Journal of Viral Hepatitis*.

[5] Lukavsky PJ (2009). Structure and function of HCV IRES domains. *Virus Research*.

[6] Suzuki T, Ishii K, Aizaki H, Wakita T (2007). Hepatitis C viral life cycle. *Advanced Drug Delivery Reviews*.

[7] El Awady MK, Azzazy HM, Fahmy AM (2009). Positional effect of mutations in 5′UTR of hepatitis C virus 4a on patients' response to therapy. *World Journal of Gastroenterology*.

[8] Otto GA, Puglisi JD (2004). The pathway of HCV IRES-mediated translation initiation. *Cell*.

[9] Hellen CU, Pestova TV (1999). Translation of hepatitis C virus RNA. *Journal of Viral Hepatitis*.

[10] Lu H, Wimmer E (1996). Poliovirus chimeras replicating under the translational control of genetic elements of hepatitis C virus reveal unusual properties of the internal ribosomal entry site of hepatitis C virus. *Proceedings of the National Academy of Sciences of the United States of America*.

[11] Laskus T, Radkowski M, Piasek A (2000). Hepatitis C virus in lymphoid cells of patients coinfected with human immunodeficiency virus type 1: evidence of active replication in monocytes/macrophages and lymphocytes. *The Journal of Infectious Diseases*.

[12] Laskus T, Radkowski M, Wang L, Nowicki M, Rakela J (2000). Uneven distribution of hepatitis C virus quasispecies in tissues from subjects with end-stage liver disease: confounding effect of viral adsorption and mounting evidence for the presence of low-level extrahepatic replication. *Journal of Virology*.

[13] Forton DM, Karayiannis P, Mahmud N, Taylor-Robinson SD, Thomas HC (2004). Identification of unique hepatitis C virus quasispecies in the central nervous system and comparative analysis of internal translational efficiency of brain, liver, and serum variants. *Journal of Virology*.

[14] Laporte J, Bain C, Maurel P, Inchauspe G, Agut H, Cahour A (2003). Differential distribution and internal translation efficiency of hepatitis C virus quasispecies present in dendritic and liver cells. *Blood*.

[15] Boyum A (1968). Separation of leukocytes from blood and bone marrow. *Scandinavian Journal of Clinical and Laboratory Investigation, Supplementum*.

[16] Laskus T, Radkowski M, Wang LU, Vargas H, Rakela J (1998). Search for hepatitis C virus extrahepatic replication sites in patients with acquired immunodeficiency syndrome: specific detection of negative- strand viral RNA in various tissues. *Hepatology*.

[17] Laskus T, Radkowski M, Jablonska J (2004). Human immunodeficiency virus facilitates infection/replication of hepatitis C virus in native human macrophages. *Blood*.

[18] Tamura K, Peterson D, Peterson N, Stecher G, Nei M, Kumar S (2011). MEGA5: molecular evolutionary genetics analysis using maximum likelihood, evolutionary distance, and maximum parsimony methods. *Molecular Biology and Evolution*.

[19] Zuker M (2003). Mfold web server for nucleic acid folding and hybridization prediction. *Nucleic Acids Research*.

[20] Thélu MA, Leroy V, Ramzan M, Dufeu-Duchesne T, Marche P, Zarski JP (2007). IRES complexity before IFN-alpha treatment and evolution of the viral load at the early stage of treatment in peripheral blood mononuclear cells from chronic hepatitis C patients. *Journal of Medical Virology*.

[21] Blackard JT, Kemmer N, Sherman KE (2006). Extrahepatic replication of HCV: insights into clinical manifestations and biological consequences. *Hepatology*.

[22] Gallegos-Orozco JF, Arenas JI, Vargas HE (2006). Selection of different 5′ untranslated region hepatitis C virus variants during post-transfusion and post-transplantation infection. *Journal of Viral Hepatitis*.

[23] Sizova DV, Kolupaeva VG, Pestova TV, Shatsky IN, Hellen CUT (1998). Specific interaction of eukaryotic translation initiation factor 3 with the 5' nontranslated regions of hepatitis C virus and classical swine fever virus RNAs. *Journal of Virology*.

[24] Sun C, Querol-Audi J, Mortimer SA (2013). Two RNA-binding motifs in eIF3 direct HCV IRES-dependent translation. *Nucleic Acids Research*.

[25] Hashem Y, des Georges A, Dhote V (2013). Hepatitis-C-virus-like internal ribosome entry sites displace eIF3 to gain access to the 40S subunit. *Nature*.

